# Decreased dopamine in striatum and difficult locomotor recovery from MPTP insult after exposure to radiofrequency electromagnetic fields

**DOI:** 10.1038/s41598-018-37874-z

**Published:** 2019-02-04

**Authors:** Ju Hwan Kim, Choong-Hyun Lee, Hyung-Gun Kim, Hak Rim Kim

**Affiliations:** 1 Department of Pharmacology, College of Medicine, Dankook University, Cheonan, Chungnam Republic of Korea; 2 Department of Pharmacy, College of Pharmacy, Dankook University, Cheonan, Chungnam Republic of Korea

## Abstract

Concern is growing about possible neuronal effects of human exposure to radiofrequency electromagnetic fields because of the increasing usage of cell phones and the close proximity of these devices to the brain when in use. We found that exposure to a radiofrequency electromagnetic field (RF-EMF) of 835 MHz (4.0 W/kg specific absorption rate [SAR] for 5 h/day for 12 weeks) affects striatal neurons in C57BL/6 mice. The number of synaptic vesicles (SVs) in striatal presynaptic boutons was significantly decreased after RF-EMF exposure. The expression levels of synapsin I and II were also significantly decreased in the striatum of the RF-EMF-exposed group. RF-EMF exposure led to a reduction in dopamine concentration in the striatum and also to a decrease in the expression of tyrosine hydroxylase in striatal neurons. Furthermore, in behavioral tests, exposure to RF-EMF impeded the recovery of locomotor activities after repeated treatments with 1-methyl-4-phenyl-1,2,3,6-tetrahydropyridine (MPTP). These results suggest that the observed decrease in dopamine concentration in the striatum was caused by both a reduction in the number of dopaminergic neurons and a decline in the number of SVs. The decreased dopamine neuron numbers and concentration seen after RF-EMF exposure would have caused the difficult recovery after MPTP treatment. In summary, our results strongly suggest that exposing the brain to RF-EMF can decrease the number of SVs and dopaminergic neurons in the striatum. These primary changes impair the recovery of locomotor activities following MPTP damage to the striatum.

## Introduction

The use of cell phones has become a universal and popular means of communication around the world. This social revolution has been accompanied by persistent concerns that exposure to the radiofrequency-electromagnetic fields (RF-EMF) emitted by cell phones has a detrimental effect on human health. Notably, in 2011, the International Agency for Research on Cancer (IARC) classified RF-EMF as a potentially carcinogenic group 2B agent and informed the public of possible risks to health resulting from mobile phone use^1^. Recently, the U.S. National Toxicology Program has conducted comprehensive studies and found high exposure to RF-EMF to be associated with cancer^2^. In addition, a possibility that RF-EMF exposure causes lesions in various organs, including brain, heart, and endocrine glands, has been suggested.

Use of a cell phone usually involves direct contact of the device with the head, and close-range exposure to the phone’s RF-EMF may affect the nervous system. Despite many controversies, evidence is accumulating for biological effects of RF-EMF exposure in the central nervous system (CNS), such as changes in blood-brain barrier permeability, homeostasis of intracellular calcium, neurotransmitters, and neuronal damage^3–7^. Moreover, RF-EMF exposure activates a diversity of intracellular events including events on the apoptotic pathway, on brain extracellular signaling pathways, and in the autophagy mechanism^8–10^. Epidemiological studies have reported headache, tremor, dizziness, loss of concentration, sleep disturbance, and cognitive dysfunction attributable to exposure to RF-EMF^11–13^. It has also been suggested that frequent use of cell phones may be associated with a risk of attention deficit hyperactivity disorder in children^14^.

Previously, we found that exposure to RF-EMF could induce changes in synaptic vesicle (SV) number and in cross-sectional areas at presynaptic terminals on cortical neurons^15^. The study implicated changes in synapsin expression in causing the SV results. SVs are small organelles nearly 40 nm diameter situated at the presynaptic terminal, and are mainly implicated in the storage, release, and secretion of neurotransmitters, which is achieved in cooperation with diverse synaptic proteins such as synapsins^16^. Synapsins are a family of abundant, SV-associated phosphoproteins and critical regulators of SV dynamics and neurotransmitter release^17,18^. Moreover, abnormal levels of synapsins in the brain are implicated in neuropsychiatric disorders such as autism^19,20^, bipolar disorder^21^, schizophrenia^21–23^, and epilepsy^19,24–27^. In transgenic animal models, a deficiency of synapsins has also been shown to result in cognitive impairments, behavioral abnormalities, and deficits in social behavior^19,23^. Therefore, the expression changes of synapsins induced by exposure to RF-EMF could affect the number and size of SVs at synaptic terminals. However, the question of whether the observed changes in SV numbers could affect the release amount of neurotransmitters has not been studied. Moreover, it is not established that such changes can cause behavioral changes in an animal model.

The striatum, a major part of the basal ganglia, receives dopaminergic input through the mesolimbic and nigrostriatal dopamine systems^28^. The striatum has a variety of functions, such as cognition, but is best known for facilitating voluntary movement; dopamine plays an important role in the organization of reward-seeking behavior and motor responses^28^. The striatum is divided into the dorsal (caudate, putamen) striatum and the ventral (nucleus accumbens) striatum^29^.

In this study, we investigated in the striatum of C57BL/6 mice the possible effects of exposure to 835-MHz (high UHF) RF-EMF at a 4.0 W/kg specific absorption rate [SAR] for 5 hours daily for 12 weeks and looked for changes in the dopaminergic neurons and terminals. Specifically, we tested whether the expression level of synapsin transcripts and proteins are altered and whether the number and size of SVs at the presynaptic terminal are altered in the striatum. Furthermore, we directly measured the level of dopamine in the striatum to test for physiological changes resulting from the changes in synapsin expression and SV trafficking. Finally, we tested for possible behavioral effects attributable to the observed decrease in dopamine concentration, changes in synapsins, and changes in SVs in striatum following RF-EMF exposure, following an MPTP challenge, because the dopaminergic projections to the striatum are essential for locomotor activities.

## Results

### Expression of synapsins I/II in striatum is reduced after RF-EMF exposure

**Figure 1:**
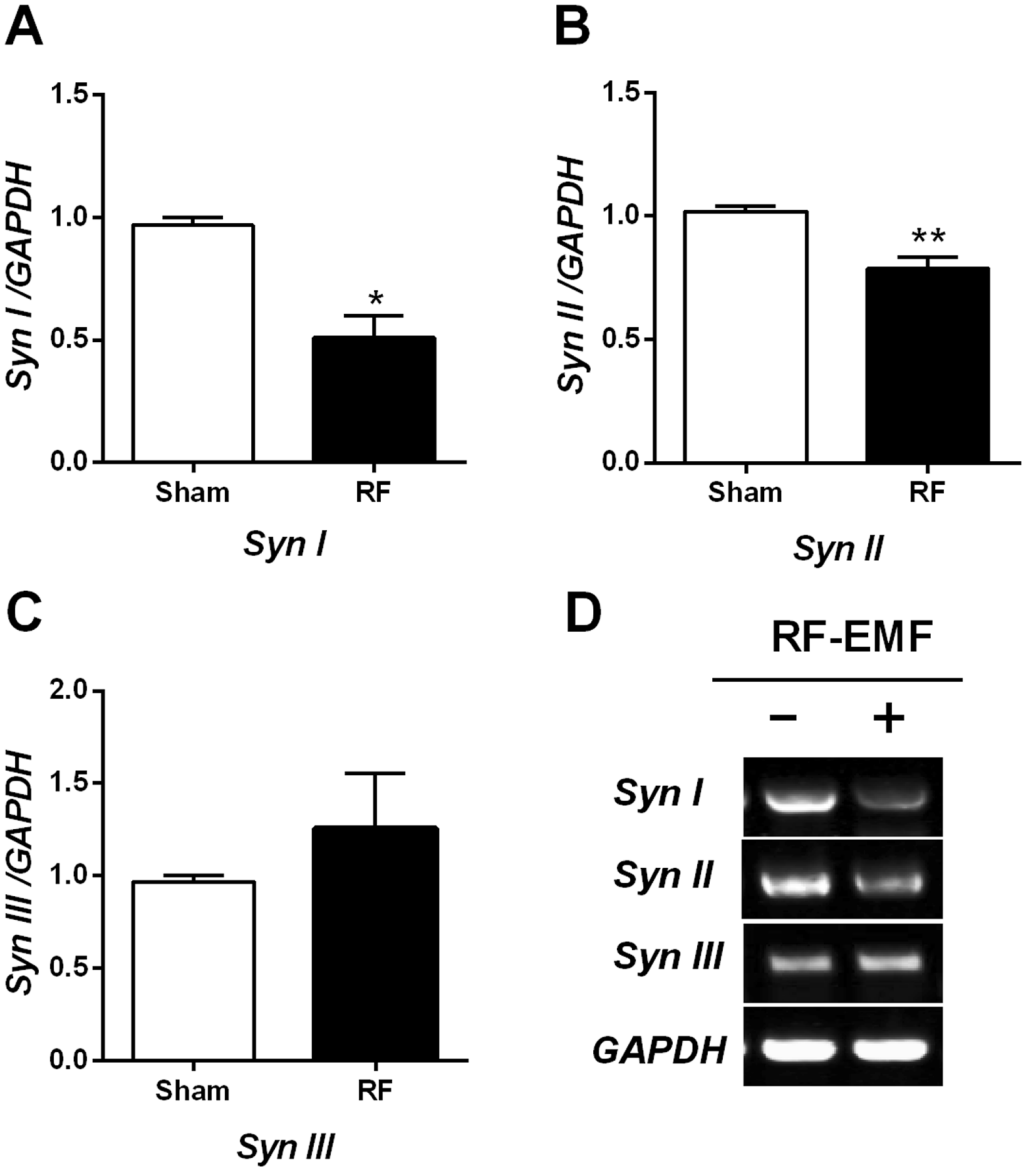
Expression levels of synapsin genes in the mouse striatum change after RF-EMF exposure. (**A**–**C**) Striatal total RNA was extracted from sham-exposed and RF-EMF-exposed mice and analyzed for synapsin I/II/III expression levels. The relative mRNA levels of synapsin I/II/III were normalized to the expression level of GAPDH by the 2^−ΔΔCt^ method. (**D**) Shown are the expression levels of synapsin genes by sqRT-PCR followed by electrophoresis on 1.5% agarose gel. Cropped, representative gel images are shown. The expression values in the striatum of RF-EMF-exposed mice were normalized to those of the sham-exposed mice. Each bar represents the mean ± SEM of three independent experiments. Statistical significance was evaluated using a two-tailed, unpaired Student’s *t*-test (*P < 0.05; **P < 0.01).

**Figure 2:**
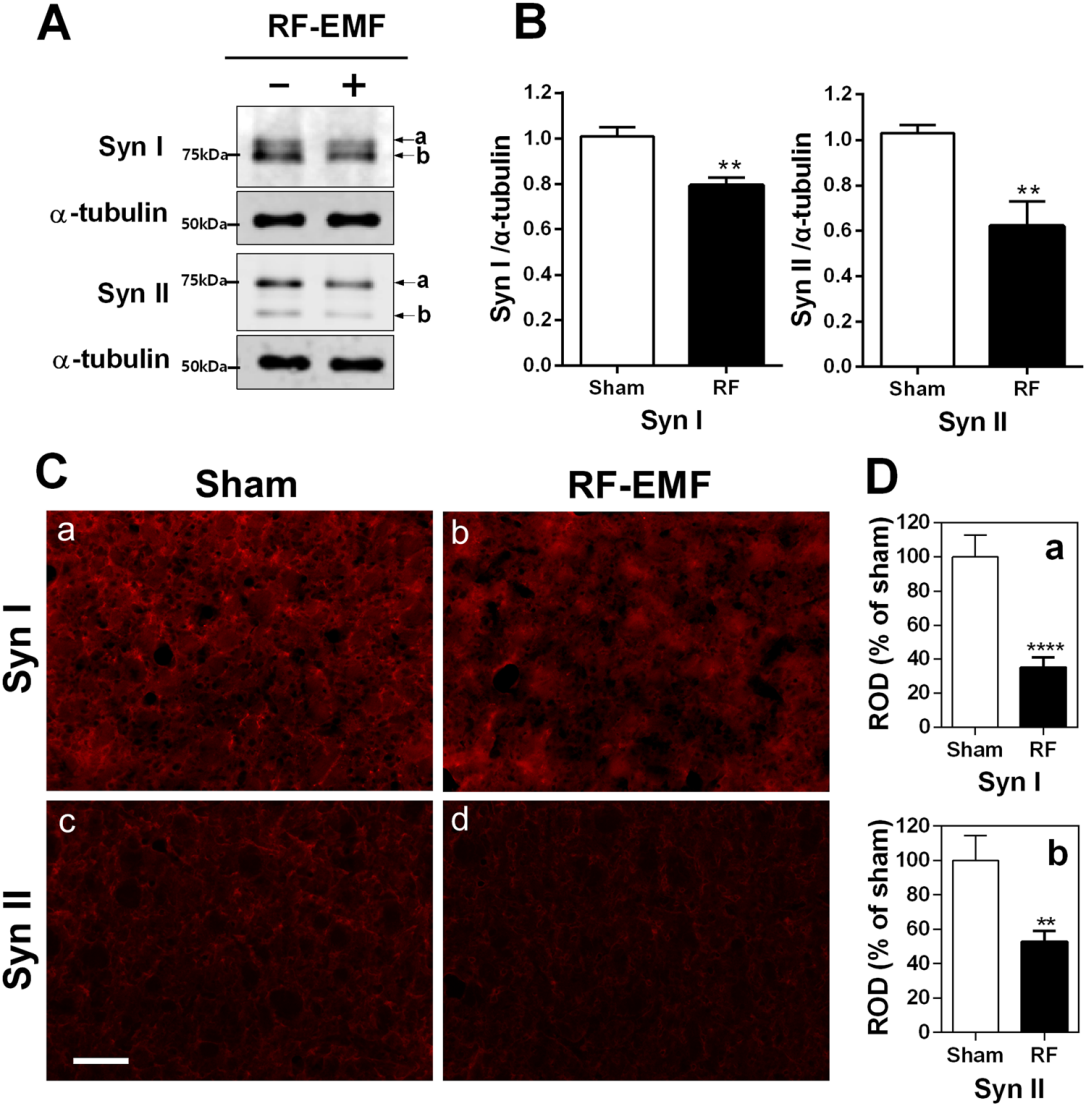
Expression levels of synapsin I/II proteins in the striatum of RF-EMF-exposed mice. (**A**) Total proteins extracted from the striatum of mice were subjected to SDS-PAGE electrophoresis and immunoblotted with antibodies against synapsin I and synapsin II. The expression levels of synapsin I/II are significantly reduced by RF-EMF exposure. Cropped, representative gel images are shown. (**B**) The protein levels of synapsin I/II were normalized relative to α-tubulin. Each bar shows the mean ± SEM of three independent experiments. (**C**) Representative immunofluorescence images of synapsin I/II in striatum. Scale bar = 100 μm. (**D**) Relative optical density (ROD). The ratio of the ROD was calibrated as %, with the sham designated as 100%. Each bar represents the mean ± SEM. Statistical significance was evaluated using two-tailed unpaired Student’s *t*-test (**P < 0.01, ****P < 0.0001).

We performed quantitative real-time polymerase chain reaction (qRT-PCR) for synapsins I/II/III in the striatum of mouse brain to elucidate whether RF-EMF exposure affects their expression level. The results indicated that the synapsin I/II mRNA level was significantly reduced in the striatum after exposure to RF-EMF for 12 weeks (Fig. 1A,B). However, the level of synapsin III was not significantly changed (Fig. 1C). Subsequently, we performed semi-quantitative PCR (sqPCR) to confirm the expression-level changes in synapsin genes. The sqPCR data showed that levels of synapsin I/II were significantly decreased but levels of synapsin III were increased, similar to the qPCR results (Fig. 1D). The RT-PCR results demonstrate an alteration of synapsin transcriptional levels in the mouse striatum following RF exposure. To confirm the RT-PCR results, we conducted western blot analysis with anti-synapsin I/II antibody (Abcam), which detects both synapsin I/IIa and I/IIb subunits. The expression levels of both synapsin I and II were significantly downregulated in the striatum following exposure (Fig. 2A,B). To validate the results of RT-PCR and immuno blot analysis, immunohistochemical analysis was conducted and indicated that both synapsin I and synapsin II immunoreactivity are markedly decreased in the RF-EMF exposed group compared to sham-exposed group (Fig. 2C). Quantitative analysis revealed that immunofluorescence staining for synapsin I and synapsin II significantly decreased more than 50% in the striatal neurons of RF-EMF exposed group (SynI: 35.22 ± 5.850, n = 14 and Syn II: 52.78 ± 6.317, n = 11) compared to sham-exposed group (SynI: 100.0 ± 12.64, n = 12 and Syn II: 100.0 ± 14.50, n = 11) (Fig. 2D). These results indicated that transcriptional and translational levels of both synapsin I and II in the striatum of mice were significantly decreased by RF-EMF exposure.

### The number and size of SVs in striatal neurons are changed by RF-EMF exposure

**Figure 3:**
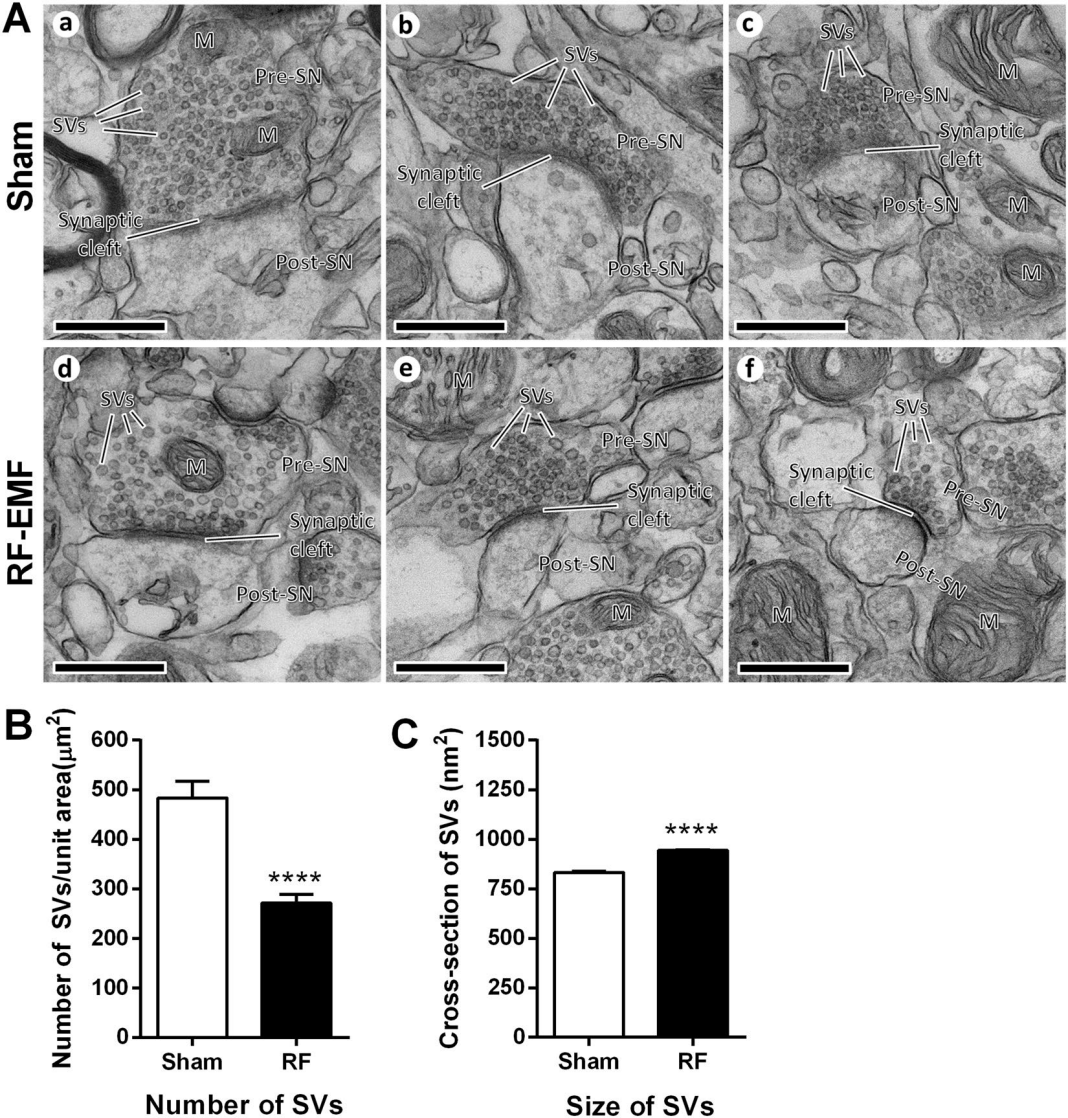
Changes in the number and size of SVs in mouse striatum after RF-EMF exposure. (**A**) Representative TEM micrographs of the synaptic region in the striatum were acquired from sham- (a, b and c) and RF-EMF-exposed (RF) mice (d, e and f), respectively. M, mitochondria; Pre-SN, pre synaptic neuron; Post-SN, post synaptic neuron; SVs, synaptic vesicles; scale bars, 500 nm. (**B,C**) Comparison of SV density and size between sham- and RF-EMF exposed mice. 30–31 synapses were randomly chosen in each condition. (**B**) Shown are numbers of SVs per square micron (**C**) Shown are the cross-sectional areas (nm^2^) of the SVs. Each bar represents the mean ± SEM. Statistical significance was evaluated using two-tailed unpaired Student’s *t*-test (****P < 0.0001).

To explore whether the decreased levels of synapsin I/II expression could affect SVs in presynaptic terminals, we acquired ultrastructure images of SVs in striatal neurons by transmission electron microscope after 835-MHz RF-EMF exposure for 12 weeks. We found no detectable structural differences in SVs between the sham-exposed and the RF-EMF-exposed group (Fig. 3A). Next, we measured the number of SVs per unit area and the size of the SVs in 30–31 randomly selected synapses in each treatment group. The results showed that the density of SVs (numbers/μm^2^) was significantly decreased (almost two-fold) in the RF-EMF-exposed group (271.41 ± 17.19) compared with the control group (483.29 ± 33.88) (Fig. 3B). However, the cross-sectional area of the SVs (nm^2^) was significantly increased in the RF-EMF exposed group (943.85 ± 9.27, from 2089 SVs) compared with the control group (832.35 ± 5.71, from 3327 SVs) (Fig. 3C).

### The dopamine concentration in the striatum is decreased by RF-EMF exposure

**Figure 4:**
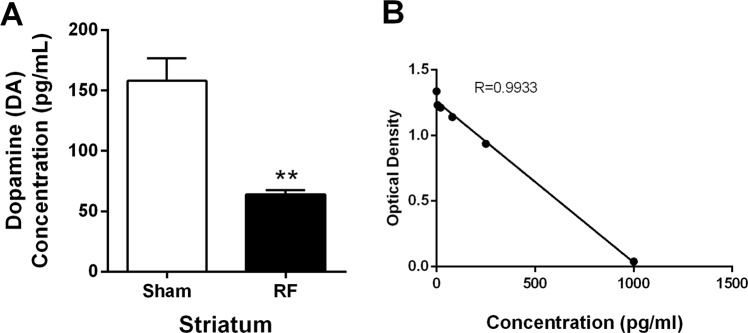
Striatal dopamine concentration decreases after RF-EMF exposure. (**A**) Shown are the measured striatal dopamine levels. (**B**) Internal calibration for dopamine analysis. Each bar represents the mean ± SEM. Statistical significance was evaluated using two-tailed, unpaired Student’s *t*-test (*P < 0.05).

Because the number and size of the SVs were changed by RF-EMF exposure, we elucidated the question of whether these changes could affect neurotransmission at the synaptic terminals. Accordingly, we measured the dopamine concentration in the striatum and found it to be significantly decreased (nearly 2.5-fold) in the RF-EMF exposed group compared with the sham group (64.11 ± 3.33 vs. 158.0 ± 18.70 pg/mL) (Fig. 4). These results suggest that dopamine neurotransmission in striatum might be decreased by RF exposure.

### The expression of tyrosine hydroxylase in the striatum is decreased by RF-EMF exposure

**Figure 5:**
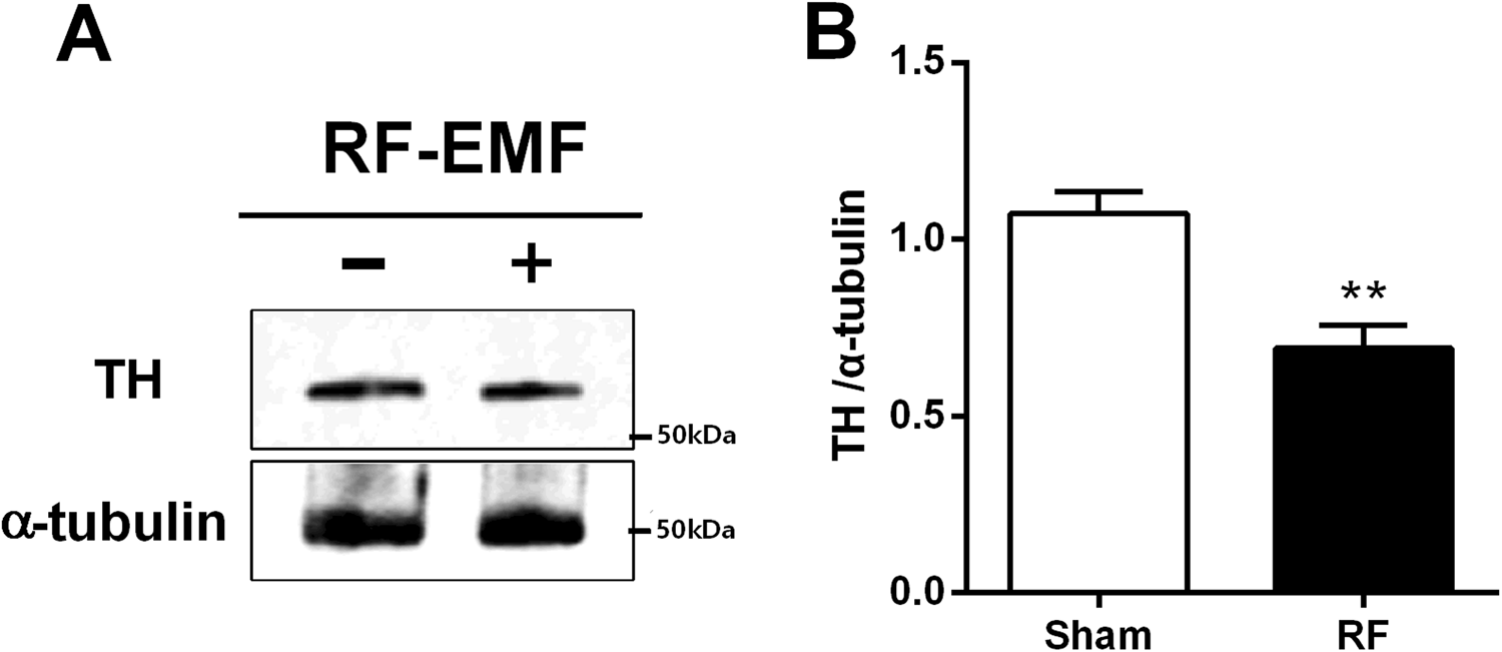
Striatal TH is down-regulated after RF-EMF exposure. (**A**) Total proteins were subjected to SDS-PAGE electrophoresis and immunoblotted with anti-TH antibody. Cropped, representative gel images are shown. (**B**) Shown is quantified band intensity. The TH protein level was normalized to α-tubulin. Each bar shows the mean ± SEM of three independent experiments. Statistical significance was evaluated using two-tailed unpaired Student’s *t*-test (**P < 0.01).

**Figure 6:**
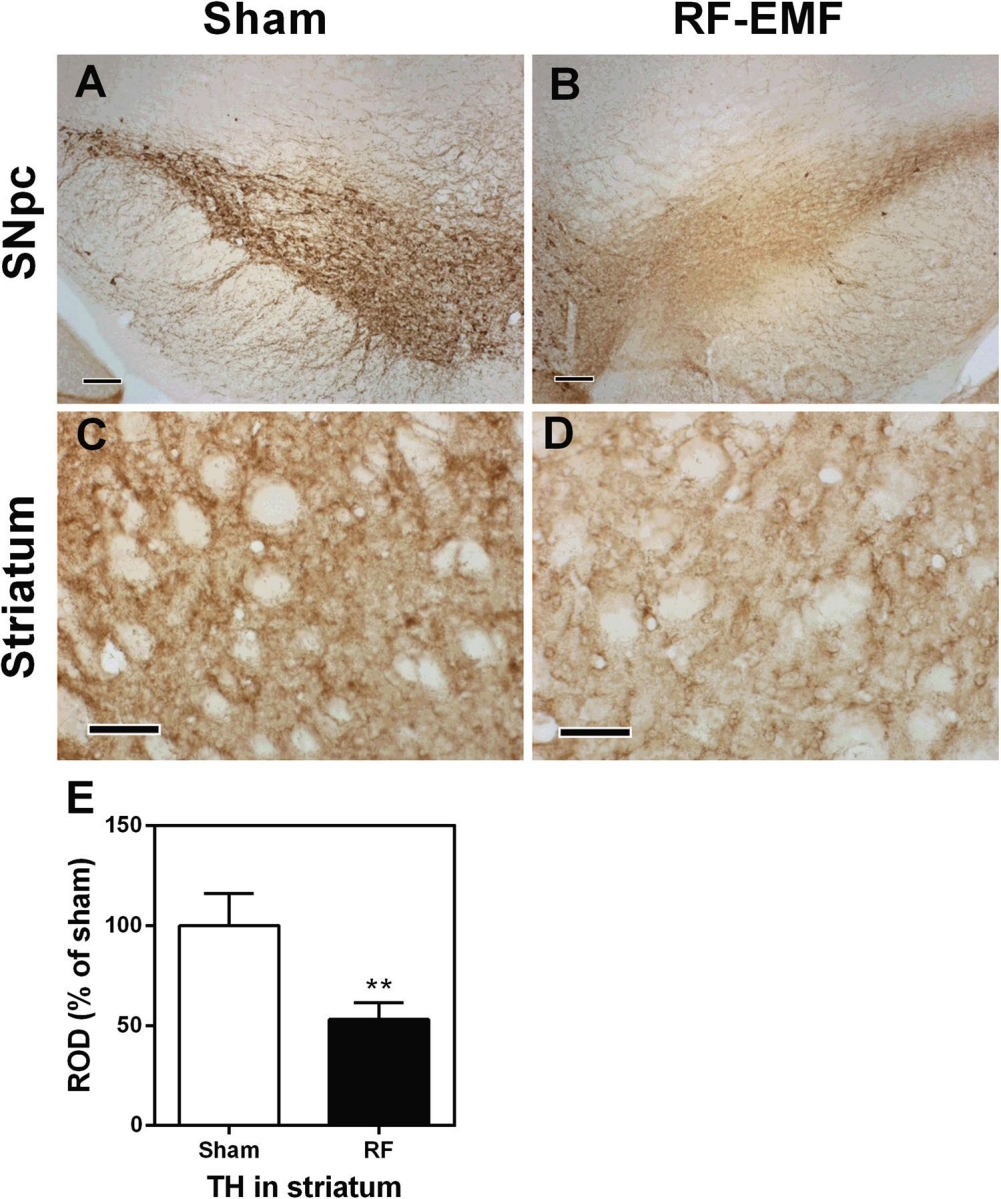
Striatal dopaminergic terminals are reduced after RF-EMF exposure. TH immunohistochemical analysis in substantia nigra pars compacta (SNpc) (**A**,**B**) and striatum (**C,D**) in sham-exposed (**A,C**) and RF-EMF exposed (**B,D**) mice. TH immunoreactivities are markedly decreased in the SNpc and striatum in the RF-EMF-exposed group. Scale bar = 100 μm. (**E**) Relative optical density (ROD) as % for TH-immunoreactive structures in the striatum in the sham- and RF-EMF groups. Bars indicate mean ± SEM. Statistical significance was evaluated using two-tailed, unpaired Student’s *t*-test (**P < 0.01; vs. sham).

To explore whether numbers of dopaminergic terminals in the striatum are affected by RF-EMF exposure, the expression of tyrosine hydroxylase (TH), a marker for dopaminergic neurons, was determined by immunoblotting and immunohistochemistry. The expression level of TH protein in the striatum was significantly decreased, by up to 30%, in the RF-EMF-exposed group (Fig. 5). In addition, immunohistochemical analysis showed that the population of TH-stained neurons/axons was significantly decreased both in the substantia nigra pars compacta (SNpc) and in the striatum of the intervention group compared with sham (Fig. 6). These results showed that the expressional level of TH was downregulated in the SNpc and striatum and suggest that the number of dopaminergic terminals in the striatum might be decreased by RF-EMF exposure.

### Exposure to RF-EMF leads to difficult locomotor recovery after MPTP challenge

To confirm that the dopamine reduction seen in the mouse striatum after RF-EMF exposure influences behavior, we measured general locomotor activities by open field test and basic motor activity by rotarod test. The behavioral tests were performed before and after challenge with MPTP (1-methyl-4-phenyl-1,2,3,6-tetrahydropyridine), which maximizes dopaminergic neuron-related motor symptoms, all after 12 weeks of exposure to RF-EMF. MPTP is a neurotoxin known to damage dopaminergic neurons specifically. The levels of TH in the striatum were significantly decreased by MPTP treatment in both sham and RF-EMF-exposed groups (Fig. S1).

**Figure 7:**
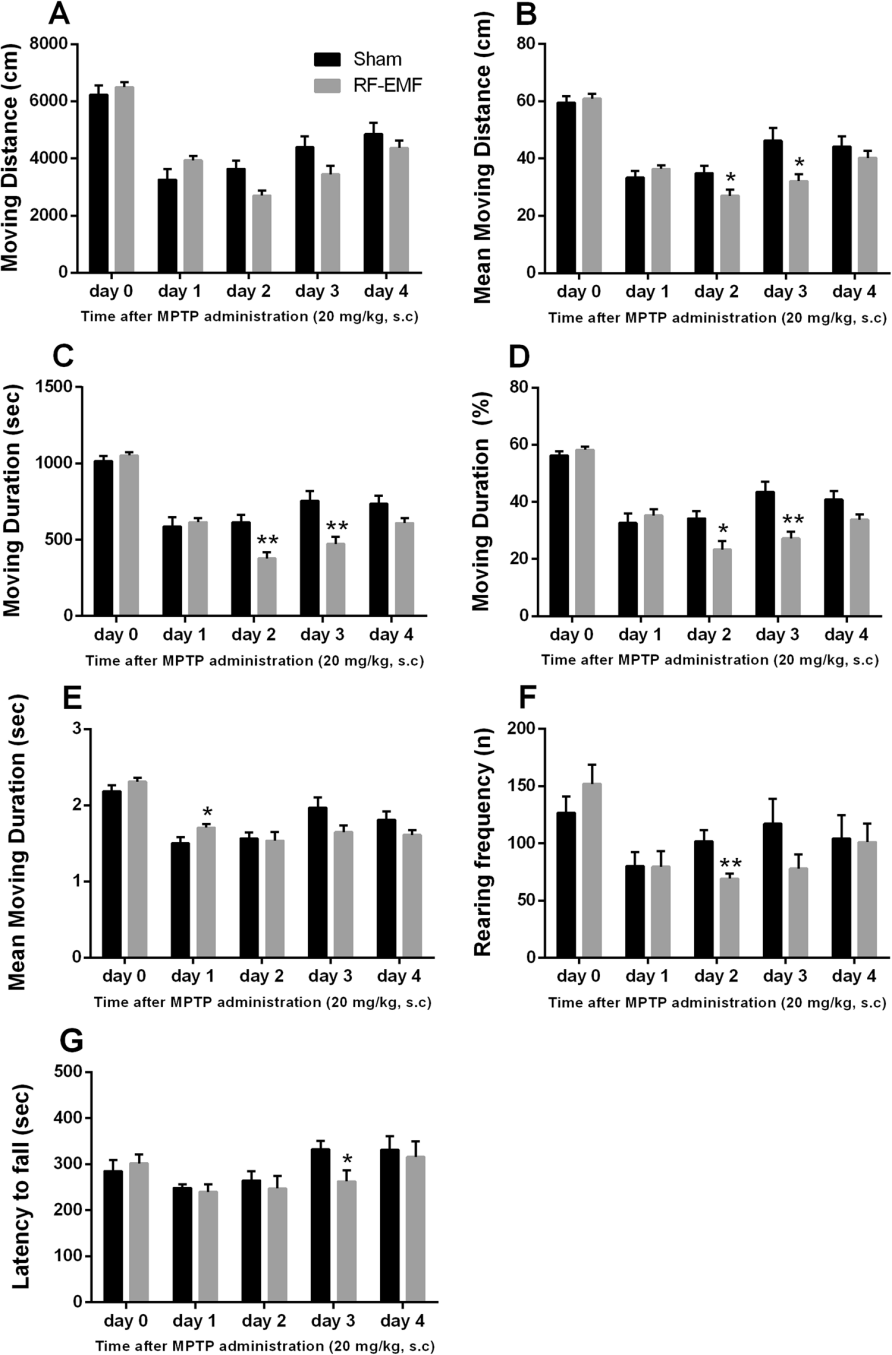
General locomotor activities and basic motor activities before and after MPTP administration. Sham-exposed and RF-EMF exposed mice were treated with 20 mg/kg MPTP for 3 days at regular intervals and examined over five days for deficits of locomotor activity and basic motor activity before/after MPTP administration. General locomotor activity measured total moving distance (**A**), mean moving distance (**B**), total moving duration (**C**), total moving duration (**D**), mean moving duration (**E**), and rearing frequency (**F**) in the open field behavioral test and basic motor activity measured latency to fall (**G**) by rotarod test. Each bar shows the mean ± SEM of 8 mice. Statistical significance was evaluated using the Student’s *t*-test (*P < 0.05; **P < 0.01; both sham vs. RF-EMF-exposed group).

The open field behavior tests indicated no significant changes, but the RF-EMF-exposed mice showed slightly higher scores for moving distance, moving duration, and rearing frequency (Fig. 7A–F). On day 1 after MPTP injection, behavior scores in the open field test decreased steeply for mice in both conditions. At day 2 following MPTP treatment, the mice showed significantly decreased moving distance, moving duration, and rearing frequency compared with sham-exposed mice (Fig. 7B–D,F) and these trends continued until day 4 (Fig. 7A–F). Sham-exposed mice showed no apparent change in their behaviors between days 1 and 2 but their behavior scores after day 3 showed large increases in moving distance, moving duration, and rearing frequency (Fig. 7A–F).

The rotarod test indicated the same overall pattern of behavior before/after MPTP injection as the open field test, but the latency-to-fall score was slightly decreased after MPTP treatment (Fig. 7G). In addition, by day 3 after MPTP injection, sham-exposed mice had completely recovered to the falling time before MPTP treatment and their falling latency was longer than that of the RF-EMF-exposed mice on day 3 after MPTP injection (*P* < 0.05; Fig. 7G).

## Discussion

In this study, we provide the first direct evidence for neurological effects of RF-EMF exposure, including changes in neurotransmitter levels in the mouse striatum and in the level of difficulty of locomotor recovery after MPTP treatment. The present findings demonstrated that 835-MHz, RF-EMF at 4.0 W/kg SAR for 5 h daily for 12 weeks resulted in alteration of neurological function in the striatum of the mouse brain, which led to a decrease in dopamine level due to decreased numbers of SVs and dopaminergic terminals in the striatum, thereby leading to increased difficulty in locomotor recovery following MPTP treatment.

The trafficking of SVs is regulated by various proteins such as synaptogyrin, synaptophysin, synaptotagmin, VAMP, SNARE, and so on^29^. However, synapsins are the key regulators of SV dynamics in presynaptic terminals^17,18^. Three kinds of synapsin are known in mammals, each with at least two isoforms^30^. The best-known function of synapsin proteins is to regulate synaptic transmission by controlling the storage and mobilization of SVs within a reserve pool (RP)^18,31^. Thus, synapsins were used here as a synaptic-vesicle marker and were found to be changed in expression level, which may affect SV morphology in the striatum after RF-EMF exposure. We previously reported that RF-EMF exposure could lead to alteration in SVs and changes in synapsin levels in cortical neurons^15^. The expression of synapsin I/II transcripts and proteins was here significantly downregulated in the striatum after RF-EMF exposure (Figs 1 and 2). Moreover, the gene profile results by microarray revealed that exposure to RF-EMF led to decreases in synapsins I/II in the striatum of mice (Table S1). However, no significant changes in synapsin III mRNA level were observed since the basal level of the synapsin III gene is very low in adult mice and is highly expressed only at the developmental stage^32^. Further, this is consistent with our microarray data, which shows that the basal level of synapsin III gene expression is more than 100 times lower than that of synapsins I/II (Table S1).

We tested whether the reduced levels of synapsin I and II seen after RF-EMF exposure could influence SV morphology in the striatum. In this study, counting the number of SVs per unit area and measuring the size of single SVs by TEM served for the morphological evaluation of SVs. The density of SVs (numbers/μm^2^) was significantly decreased, but the size of the SVs was significantly increased in striatal neurons in the RF-EMF exposed group (Fig. 3). The increased size of SVs may have partly compensated for the reduced number of SVs. This is consistent with other published results showing that lack of synapsin I and/or II in mice decreases the number of SVs in hippocampal, spinal, and visual cortex neurons^27,33,34^ but in parts of the forebrain, the SVs are larger in size^35^. Moreover, synapsins interact with SV proteins and phospholipids and play an important role in the regulation of SV trafficking and stability by cross-linking SVs to each other and to the actin cytoskeleton. The membranes of SVs are mostly made of phospholipids, which form a bilayer, and are normally coated with synapsins, especially synapsin I^35^. Therefore, the normal SV is tightly wrapped in synapsins. However, it was reported that synapsin-depleted SVs in the rat forebrain became larger in size, i.e., a spherical shape but with a diameter of up to 70 nm vs. the typical diameter of 40 nm, due to the absence of synapsin I surrounding the phospholipid bilayer^35^. As shown in our data, the expression levels of synapsin genes and proteins were reduced by exposure to RF-EMF, which may account for the observed larger size. These results suggested that deceased levels of synapsins directly affect SV stability, reducing the number of SVs seen in the striatum in the RF-EMF-exposed group. We conclude that RF-EMF exposure leads to a decrease in the number of SVs in the striatum via reductions in the expression of synaptic proteins such as synapsin I and II.

The striatum is a subcortical area of the forebrain and the major input area of the basal ganglia, and receives dopaminergic input through the mesolimbic and nigrostriatal dopamine systems^28^. Interestingly, the present study showed that the level of dopamine dramatically decreased in the mouse striatum following RF-EMF exposure for 12 weeks (Fig. 4). Previously, it had been reported that low-level, extremely low frequency (ELF)-EMFs at 1000 milliGauss for 1 month caused slight alterations in levels of neurotransmitters, including dopamine, in the hypothalamus and striatum of rats^36^. Moreover, exposure to 1800 MHz RF-EMF with a 0.843 W/kg SAR resulted in changes in monoamines such as dopamine, NE, and 5-HT in the hippocampus, hypothalamus, midbrain, and medulla oblongata of the rat brain^37^. These findings are also consistent with a preliminary result by our research group that the dopamine level decreased by about 25% in the mouse striatum after RF-EMF exposure for 4 weeks, as shown by LC-MS/MS analysis (data not shown).

Of interest, the levels of TH expression in the striatum were also significantly decreased in the RF-EMF exposed group (Fig. 5). Additionally, the TH immunohistochemical study showed that the proportions of TH-immunoreactive neurons in the SNpc and striatum were significantly decreased in the RF-EMF exposed group compared with the sham-exposed group (Fig. 6). TH is the rate-limiting enzyme for dopamine synthesis and a marker of dopaminergic neurons^38^. Moreover, TH expression in dopaminergic neurons directly influences the quantitative regulation of dopamine in dopaminergic neurons, and hence TH expression can be indicative of the survival as well as the functional status of dopaminergic neurons^39^. Thus, a deficiency in TH causes impaired synthesis of dopamine^40^. Our results strongly suggest that the significant reduction in striatal dopamine concentration we saw is attributable the decreased number of SVs and the reduced population of dopaminergic neurons in the striatum we found after RF-EMF exposure.

Previously, we had reported that exposing mice to RF-EMF could cause demyelination of cortical neurons and hyperactivity on behavioral testing^41^. In this study, we further investigated the behavioral changes in the RF-EMF exposed group because we found the dopamine concentration to be decreased in the striatum by RF-EMF exposure. To maximize dopaminergic-neuron related motor behaviors, behavioral tests were combined with challenge by MPTP, which destroys dopaminergic neurons in the SNpc and the striatum and finally leads to a Parkinsonian syndrome^42–44^ involving slow movements, tremors, and rigidity^45,46^. As previously reported^41^, behavioral analysis showed hyperactivity in the RF-EMF-exposed group before MPTP treatment (day 0), representing increases in general locomotor and motor activities (Fig. 7). Although we found no significant differences in moving distance, the mean moving distance was significantly decreased in the RF-EMF-exposed group at day 2–3 after MPTP treatment (Fig. 7A,B). The moving distance is the total length moved in a given time and the mean moving distance is the average distance from the start point to the stop point after 30 min. In addition, RF-EMF-exposed mice exhibited lessened moving durations compared with sham after MPTP treatment (Fig. 7C,D). Overall, the general locomotor and basic motor activities were significantly lower in the RF-EMF-exposed group at day 2–3 after MPTP administration (Fig. 7). These data indicated that behavioral recovery from acute damage to dopaminergic neurons was more difficult in the RF-EMF-exposed group because of reduced numbers of SVs in the striatum. Overall, the mice exposed to RF-EMF before MPTP administration were hyperactive, their locomotor activity was sharply reduced after MPTP treatment, and then gradually recovered. In contrast, the behavioral activities of the sham group started to recover from MPTP injection sooner and maintained a higher level of moving distance and duration, rearing frequency, and falling latency by day 4 than the RF-EMF-exposed group. These results suggest that the recovery processes of striatal dopaminergic neurons damaged by MPTP administration are more difficult or delayed in RF-EMF-exposed mice due to additional reductions in SVs at striatal neurons.

In summary, exposure to 4 W/kg SAR, 835 MHz RF-EMF for 12 weeks leads to decreased numbers of SVs associated with a reduced level of synapsin I/II in presynaptic terminals as well as a reduced level of TH expression in striatum, thereby decreasing the dopamine level in the striatum, and eventually these changes might lead to behavioral alterations. Therefore, the observed changes in striatal dopaminergic neurons induced by exposure to RF-EMF might influence locomotor activity under conditions of impaired neuronal function.

## Methods

### Mice

Six-week-old C57BL/6 mice (weighing 25–30 g, male) were purchased from Daehan Bio Link (Chungbuk, South Korea) and maintained under controlled conditions (an ambient temperature of 23 ± 2 °C with 12-h light/dark cycles). Food pellets (Daehan Bio Link) and water were supplied *ad libitum*. After a week’s adaptation period, mice were randomly assigned to groups as described below. All mice procedures adhered to the National Institutes of Health Guidelines for Animal Research and were approved by Dankook University Institutional Animal Care and Use Committee (IACUC; DKU-15-001), which adheres to the guidelines issued by the Institute for Laboratory Animal Research.

### RF-EMF exposure

Mice were exposed to 835 MHz RF-EMF using a Wave Exposer V20, which has already been tested regarding dosimetry (i.e., measuring spectrum (MHz) or body-average specific absorption rate (SAR) value) for our RF-EMF generator as described in detail^41^. Whole body exposure was at SAR value of 4.0 W/kg for 5 h daily during 12 weeks for randomly allocated mice. The remaining mice received sham treatment for 12 weeks. The sham-treated group was kept under identical environmental conditions and treated with the same circular pattern as the RF-exposed groups without the RF-EMF exposure. The sham-treated and RF-exposed mice could move freely in their cages. The size of the mouse cage inside the RF-EMF generator was 43 cm L × 37 cm W × 18 cm H. RF-EMF exposure involved a top horn antenna equipped at the lower mouse cage. The bottom and wall of the cage were covered in ceramic wave absorption material. The intent was to mimic RF with SAR exposure in the open environment to exclude the possibility of on the number of mice altering exposure. Moreover, the RF exposure apparatus was equipped with an automatic light system, air conditioning, and water dispenser. The mice were not restricted in movement in the cage during exposure. All the experiments were performed in our animal facility and were conducted at a constant temperature. To exclude the possibility of thermal effects on mice from our RF-EMF generator, mouse body temperature was measured before and after RF-EMF exposure, indicating that exposure to RF-EMF in freely moving mice did not affect mouse body temperature during the 5 h of exposure to 835 MHz RF-EMF at 4.0 W/kg SAR emitted from our RF-EMF generator^41^.

### Reverse transcription and quantitative real-time polymerase chain reaction (PCR)

Total RNA was purified using TRIzol reagent (Thermo Fisher Scientific, Pittsburgh, PA) from the striatum of mice (n = 10). RNA was reverse transcribed to cDNA using MMLV reverse transcriptase (Bioneer, Daejeon, South Korea) and an oligo-d(T)18 primer. Quantitative real time PCR (qRT-PCR) was performed using a Rotor Gene SYBR Green supermix Kit (QIAgen, Hilden, Germany) and fluorescence was measured using a Rotor-gene PCR Cycler (QIAgen). Glyceraldehyde 3-phosphate dehydrogenase (GAPDH) was used as a housekeeping gene. The primers were synthesized from Bioneer. Oligo-nucleotide sequences of primers used for sqRT-PCR and qRT-PCR. The sequences for forward and reverse synapsin primers were used: Syn I F: 5′- CAGGGTCAAGGCCGCCAGTC-3′ and R: 5′-CACATCCTGGCTGGGTTTCTG-3′; Syn II F: 5′-AGGGGAGAAATTCCCAC-3′ and R: 5′-CCCAGAGCTTGTACCG-3′; Syn III F: 5′-CCAACAGCGACTCTCG-3′ and R: 5′-GGTTGCGGATTGTCTC-3′ ^47^. GAPDH primer was purchased from QIAgen. Three biologically independent experiments were performed and each PCR reaction was repeated in triplicate. The relative levels of specific mRNA were calculated by normalizing to the expression of GAPDH by the 2^−ΔΔCt^ method. Furthermore, the expression values of the RF-EMF exposed group were normalized to those of the sham-exposed group. Semi-quantitative RT-PCR (sqRT-PCR) reactions were performed with PCR PreMix (Bioneer, Daejeon, South Korea). The obtained PCR product of each gene was used for 1.5% agarose gel electrophoresis and the signal intensity of each product was visualized using Syto 60 (Li-COR Biosciences, Lincoln, NB) stained DNA and an Odyssey infrared imaging system (Li-COR Biosciences).

### Immunoblotting

Sham-exposed or RF-EMF-exposed mice (n = 10) were quickly sacrificed and the striatum was rapidly dissected from the mouse brain. The tissue was lysed with RIPA buffer (ATTO, Tokyo, Japan) supplemented with protease and phosphate inhibitor cocktail (ATTO). Whole lysates were then homogenized in ice-cold buffer and sonicated briefly. Protein concentrations were measured using a Bio-Rad DC^TM^ protein assay (Bio-Rad, Hercules, CA) and total protein (20–50 μg) were separated by 10% sodium dodecyl sulfate-polyacrylamide gel electrophoresis and transferred with transfer buffer to a polyvinylidene difluoride (PVDF) transfer membrane (ATTO). Synapsin I, synapsin II, tyrosine hydroxylase (TH), and α-tubulin were detected in the membranes using anti-Synapsin I antibody (1:1000, Abcam, Cambridge, MA #ab64581), anti-Synapsin II antibody (1:3000, Abcam #ab76494), anti-TH antibody (1:500, Sigma-Aldrich, St. Louis, MI #T2928), and anti-α-tubulin (1:3000, Santa Cruz Biotechnology, Santa Cruz, CA #sc-23948). Protein bands were visualized using Odyssey infrared imaging system (Li-Cor Biosciences). The intensity of each band was quantified and normalized using α-tubulin as an internal loading control.

### Transmission electron microscopy (TEM)

The dissected striatum from the mice of each condition (n = 3) was immediately fixed in 2% glutaraldehyde and 2% paraformaldehyde in 0.1 M phosphate buffer (pH 7.4) for 2 h at 4 °C. Following three washes in phosphate buffer, the brain tissues were post-fixed with 1% osmium tetroxide on ice for 2 h and washed three times, all in phosphate buffer. The tissues were then embedded in Epon 812 after dehydration in ethanol and propylene oxide series. Polymerization was conducted with pure resin at 70 °C for 24 h. Ultrathin sections (~70 nm) were obtained with a model MT-X ultramicrotome (RMC, Tucson, AZ,) and then collected on 100 mesh copper grids. After staining with 2% uranyl acetate (15 min) and lead citrate (5 min), the sections were visualized by a high performance, high contrast, 120 kV TEM (JEM-1400 Plus, JEOL, Japan).

### Measurements for number and size of SVs

Samples were immediately prepared with control mice group (n = 3) and RF-EMF exposed mice group (n = 3). We obtained 10–11 synaptic images per mouse and counted synaptic vesicles (SVs) in 31 control group and 30 synapses RF-EMF exposed group. Moreover, the area of synaptic vesicles (SVs) in all pre-synapse used for counting SV was measured. However, we simply selected only the SV membrane which was clearly distinguished and then diameter of selected 3327 SVs in control group and 2089 SVs in RF-EMF exposed group was measured. The number of SVs per unit area (μm^2^) and the cross-sectional area of SVs (nm^2^) was obtained according to the instructions of the previous report^15^.

### Immunohistochemistry for tyrosine hydroxylase

For immunohistochemical analysis, the animals (n = 3) were anesthetized with zoletil 50 (30 mg/kg, Virbac, Carros, France) and perfused transcardially with 0.1 M phosphate-buffered saline (PBS) followed by 4% paraformaldehyde in 0.1 M PBS. The brain tissues were removed, cryoprotected, and serially sectioned on a cryostat (Leica, Wetzlar, Germany) into 30-μm coronal sections, and they were then collected into six-well plates containing PBS. According to the method previously described^48^, immunohistochemistry for TH was performed with rabbit anti-tyrosine hydroxylase (1:500 #AB152, Millipore, Bedford, MA) as a primary antibody. In order to establish the specificity of the immunostaining, a negative control test was performed without a primary antibody. In order to establish the specificity of the immunostaining, negative control tests were performed with only the secondary antibody. The negative control resulted in the absence of immunoreactivity in any structure. To analyze TH immunoreactivity, four sections per animal were selected. Digital images of the striatum were captured with an Axio Imager 2 microscope (Carl Zeiss, Germany) equipped with a digital camera (Axiocam, Carl Zeiss) connected to a PC monitor. According to the method previously described^49^, the images were calibrated into an array of 512 × 512 pixels corresponding to a tissue area of 140 × 140 μm (40 × primary magnification). The densities of TH-immunoreactive structures in the substantia nigra pars compacta (SNpc) and the striatum were evaluated on the basis of optical density (OD), which was obtained after the transformation of the mean gray level using the formula: OD = log (256/mean gray level). The OD of the background was acquired from areas adjacent to the measured area. After the background density was subtracted, a ratio of the optical density of the image file was calibrated as % (relative optical density, ROD) and analyzed using NIH Image 1.59 software. A ratio of the ROD was calibrated as %, with control-group designated as 100%.

### Immunofluorescence staining for synapsin I/II

For immunofluorescence staining for synapsin I and II, the sections of the animals (n = 3) were incubated with a rabbit anti-synapsin I (1:50, Abcam #ab64581) or a rabbit anti-synapsin II (1:50, Abcam #ab76494) overnight at room temperature. Then, the sections were incubated with a Cy3-conjugated goat anti-rabbit IgG (1:200; Jackson ImmunoResearch, West Grove, PA) for 2 h at room temperature. Immunofluorescence images were captured with an Axio Imager 2 microscope (Carl Zeiss). The densities of Synapsin I/II-immunoreactive structures were evaluated on the basis of OD, which was obtained after the transformation of the mean red level using the formula: OD = log (256/mean red level). The OD of background was taken from areas adjacent to the measured area. After the background density was subtracted, a ratio of the optical density of image file was calibrated as % (relative optical density, ROD) and analyzed using NIH Image 1.59 software. A ratio of the ROD was calibrated as %, with control-group designated as 100%.

### Measurements of dopamine in the striatum

Experimental mice (n = 3) were euthanized by cervical dislocation and the head was rapidly removed with scissors, then the striatum was rapidly dissected from each brain on ice. Dopamine concentration was determined in the striatal tissue of either sham-exposed or RF-EMF-exposed mice (n = 3) using the mouse dopamine enzyme-linked immuno specific assay (ELISA) kit (Cusabio Biotech, Wuhan, China), following the manufacturer’s instructions. The absorbance of each well was measured at a wavelength of 450 nm using a 96-well microplate spectrophotometer (Multiskan GO; Thermo Scientific, Waltham, MA). This assay can detect mouse dopamine in the range of 5–1000 pg/ml.

### MPTP challenge

Twelve-week sham- and RF-EMF-exposed mice (n = 8) were injected subcutaneously (s.c.) with MPTP (20 mg/kg). After testing with the rotarod and open field test systems as detailed below (Fig. S2), mice were euthanized by cervical dislocation, and each brain was quickly retrieved. The striatum was quickly dissected on ice under a dissecting microscope. The dissected brain regions were quick-frozen in a deep-freezer (−80 °C) until further analysis.

### Open field test

Mice (n = 8) were placed in nine plastic rectangular boxes (30 cm L × 30 cm W × 40 cm H) before/after s.c. administration of 20 mg/kg MPTP after 12 weeks of exposure to RF-EMF. Mice injected with the same volume of saline were used as the control group. All parameters were set in the open field apparatus background space (30 cm W × 30 cm L), and the arena was set in the software. In this area, the center point of the mouse body was recognized and the movement was automatically calculated and expressed as a numerical value^50–53^. The mice were observed with a CCD camera connected to a recording system. The recorded behavior was analyzed with EthoVision Version 2.3 (Noldus information Technology, Wageningen, The Netherlands,). An automated video tracking system was used for calculation of moving distances/durations and rearing frequencies. Locomotor activity of mice was observed for 30 min in normal lighting. The distance moved is the distance from the start of the movement to the end of the movement at the center of the body^51,52^. The total moving distance moved is the total length of the moving body center point in the arena for a test time (30 min)^51^. Mean moving distance is the average distance that the sum of total distance from the start point to the stop point divided by the number of movements for 30 minutes^52^. The moving duration (sec.) is the sum of all the times that movement was detected during the test time for 30 min^51,52^. The moving duration (%) is the percentage of the total time that movement is detected for 30 minutes when the total test time (30 min) is 100%. The mean moving duration (sec.) is the average moving time that the sum of total time from the start point to the stop point divided by the number of movements for 30 minutes^51,52^. The test box was cleaned with 70% alcohol and water between trials to remove any substances that could affect mouse behavioral patterns in the next test or a subsequent round of testing.

### Rotarod test

We examined the experimental mice with the rotarod behavioral test after 12 weeks of exposure to RF-EMF with eight sham-exposed mice and eight RF-EMF exposed mice before/after MPTP treatment. Mice were evaluated for their ability to stay on a rotarod (Ugo Basile, Comerio VA, Italy) for at least 600 s (16 rpm, accelerated speed). Rotarod testing was performed three times per mouse with an intervening interval of at least 10 min. The times in s from the three tests were averaged.

### Statistics

All data are presented as the mean ± SEM. The n values represent the number of independent animal samples used in the experiments. The significance for all pairwise comparisons of interest was assessed by two-tailed Student’s *t*-test with probability values of p < 0.05 considered to be significant. GraphPad Prism 4 software (GraphPad Software, La Jolla, CA) was used for the statistical analysis.

## Supplementary information

Decreased dopamine in striatum and difficult locomotor recovery from MPTP insult after exposure to radiofrequency electromagnetic fields

## References

[1] Baan R (2011). Carcinogenicity of radiofrequency electromagnetic fields. Lancet Oncol.

[2] Wyde, M. *et al*. Report of Partial findings from the National Toxicology Program Carcinogenesis Studies of Cell Phone Radiofrequency Radiation in Hsd: Sprague Dawley® SD rats (Whole Body Exposure). *bioRxiv*, 10.1101/055699 (2018).

[3] Salford LG, Brun AE, Eberhardt JL, Malmgren L, Persson BRR (2003). Nerve Cell Damage in Mammalian Brain after Exposure to Microwaves from GSM Mobile Phones. Environ Health Persp.

[4] Mausset-Bonnefont AL (2004). Acute exposure to GSM 900-MHz electromagnetic fields induces glial reactivity and biochemical modifications in the rat brain. Neurobiol Dis.

[5] Bas O, Odaci E, Mollaoglu H, Ucok K, Kaplan S (2009). Chronic prenatal exposure to the 900 megahertz electromagnetic field induces pyramidal cell loss in the hippocampus of newborn rats. Toxicol Ind Health.

[6] Nittby H (2009). Increased blood-brain barrier permeability in mammalian brain 7 days after exposure to the radiation from a GSM-900 mobile phone. Pathophysiology.

[7] Maskey D (2010). Effect of 835 MHz radiofrequency radiation exposure on calcium binding proteins in the hippocampus of the mouse brain. Brain Res.

[8] Tang J (2015). Exposure to 900 MHz electromagnetic fields activates the mkp-1/ERK pathway and causes blood-brain barrier damage and cognitive impairment in rats. Brain Res.

[9] Kim JH, Huh YH, Kim HR (2016). Induction of Autophagy in the Striatum and Hypothalamus of Mice after 835 MHz Radiofrequency Exposure. PloS One.

[10] Kim JH, Yu DH, Kim HR (2017). Activation of autophagy at cerebral cortex and apoptosis at brainstem are differential responses to 835 MHz RF-EMF exposure. Korean J Physiol Pharmacol.

[11] Santini, R., Santini, P., Danze, J., Le Ruz, P. & Seigne, M. Study of the health of people living in the vicinity of mobile phone base stations, Influences of distance and sex. *Pathol Biol* **50**, 369–373, PMID: 12168254 (2002). 10.1016/s0369-8114(02)00311-5 12168254

[12] Hutter HP, Moshammer H, Wallner P, Kundi M (2006). Subjective symptoms, sleeping problems, and cognitive performance in subjects living near mobile phone base stations. Occup Environ Med.

[13] Abdel-Rassoul G (2007). Neurobehavioral effects among inhabitants around mobile phone base stations. Neurotoxicology.

[14] Byun YH (2013). Mobile phone use, blood lead levels, and attention deficit hyperactivity symptoms in children: a longitudinal study. PloS One.

[15] Kim JH (2017). Changes in numbers and size of synaptic vesicles of cortical neurons induced by exposure to 835 MHz radiofrequency-electromagnetic field. PloS One.

[16] Sudhof TC (2004). The synaptic vesicle cycle. Annu Rev Neurosci.

[17] Gitler D, Cheng Q, Greengard P, Augustine GJ (2008). Synapsin IIa controls the reserve pool of glutamatergic synaptic vesicles. J Neurosci.

[18] Hilfiker S (1999). Synapsins as regulators of neurotransmitter release. Philos Trans R Soc Lond B Biol Sci.

[19] Greco B (2013). Autism-related behavioral abnormalities in synapsin knockout mice. Behav Brain Res.

[20] Medrihan L (2013). Synapsin II desynchronizes neurotransmitter release at inhibitory synapses by interacting with presynaptic calcium channels. Nat Commun.

[21] Vawter MP (2002). Reduction of synapsin in the hippocampus of patients with bipolar disorder and schizophrenia. Mol Psychiatry.

[22] Chong VZ, Skoblenick K, Morin F, Xu Y, Mishra RK (2006). Dopamine-D1 and -D2 receptors differentially regulate synapsin II expression in the rat brain. Neuroscience.

[23] Dyck BA (2012). Behavioral effects of non-viral mediated RNA interference of synapsin II in the medial prefrontal cortex of the rat. Schizophr Res.

[24] Boido D (2010). Cortico-hippocampal hyperexcitability in synapsin I/II/III knockout mice: age-dependency and response to the antiepileptic drug levetiracetam. Neuroscience.

[25] Ketzef M (2011). Compensatory network alterations upon onset of epilepsy in synapsin triple knock-out mice. Neuroscience.

[26] Corradi A (2008). Synapsin-I-and synapsin-II-null mice display an increased age-dependent cognitive impairment. J Cell Sci.

[27] Li L (1995). Impairment of synaptic vesicle clustering and of synaptic transmission, and increased seizure propensity, in synapsin I-deficient mice. Proc Natl Acad Sci USA.

[28] Cachope R, Cheer JF (2014). Local control of striatal dopamine release. Front Behav Neurosci.

[29] Brachya G, Yanay C, Linial M (2006). Synaptic proteins as multi-sensor devices of neurotransmission. BMC Neurosci.

[30] Porton B, Wetsel WC (2007). Reduction of synapsin III in the prefrontal cortex of individuals with schizophrenia. Schizophr Res.

[31] Song SH, Augustine GJS (2015). Isoforms and Synaptic Vesicle Trafficking. Mol Cells.

[32] Piccini A, Perlini LE, Cancedda L, Benfenati F, Giovedi S (2015). Phosphorylation by PKA and Cdk5 Mediates the Early Effects of Synapsin III in Neuronal Morphological Maturation. J Neurosci.

[33] Rosahl TW (1995). Essential functions of synapsins I and II in synaptic vesicle regulation. Nature.

[34] Takei Y (1995). Synapsin I deficiency results in the structural change in the presynaptic terminals in the murine nervous system. J Cell Biol.

[35] Awizio AK, Onofri F, Benfenati F, Bonaccurso E (2007). Influence of synapsin I on synaptic vesicles: an analysis by force-volume mode of the atomic force microscope and dynamic light scattering. Biophys J.

[36] Chance W (1995). Effects of electromagnetic fields and gender on neurotransmitters and amino acids in rats. Physiol Behav.

[37] Ezz HA, Khadrawy Y, Ahmed N, Radwan N, El Bakry M (2013). The effect of pulsed electromagnetic radiation from mobile phone on the levels of monoamine neurotransmitters in four different areas of rat brain. Eur Rev Med Pharmacol Sci.

[38] Daubner SC, Le T, Wang S (2011). Tyrosine hydroxylase and regulation of dopamine synthesis. Arch Biochem Biophys.

[39] Gao J (2011). Influence of aging on the dopaminergic neurons in the substantia nigra pars compacta of rats. Curr Aging Sci.

[40] Haavik J, Toska K (1998). Tyrosine hydroxylase and Parkinson’s disease. Mol Neurobiol.

[41] Kim JH (2017). Long-term exposure to 835 MHz RF-EMF induces hyperactivity, autophagy and demyelination in the cortical neurons of mice. Sci Rep.

[42] Bové J, Prou D, Perier C, Przedborski S (2005). Toxin-induced models of Parkinson’s disease. NeuroRx.

[43] Tieu K (2011). A guide to neurotoxic animal models of Parkinson’s disease. Cold Spring Harb Perspect Med.

[44] Lee KS, Lee JK, Kim HG, Kim HR (2013). Differential Effects of 1-methyl-4-phenyl-1,2,3,6-tetrahydropyridine on Motor Behavior and Dopamine Levels at Brain Regions in Three Different Mouse Strains. Korean J Physiol Pharmacol.

[45] Surmeier DJ, Graves SM, Shen W (2014). Dopaminergic modulation of striatal networks in health and Parkinson’s disease. Curr Opin Neurobiol.

[46] Yager LM, Garcia AF, Wunsch AM, Ferguson SM (2015). The ins and outs of the striatum: role in drug addiction. Neuroscience.

[47] Frederikse PH (2004). Synapsin and synaptic vesicle protein expression during embryonic and post-natal lens fiber cell differentiation. Mol Vis.

[48] Klionsky DJea (2016). Guidelines for the use and interpretation of assays for monitoring autophagy (3rd edition). Autophagy.

[49] Lee T-K (2016). Pretreated duloxetine protects hippocampal CA1 pyramidal neurons from ischemia-reperfusion injury through decreases of glial activation and oxidative stress. J Neurol Sci.

[50] Noldus LPJJ, Spink AJ, Tegelenbosch RAJ (2002). Computerised video tracking, movement analysis and behaviour recognition in insects. Comput Electron Agric.

[51] Tatem, K. S. *et al*. Behavioral and locomotor measurements using an open field activity monitoring system for skeletal muscle diseases. *J Vis Exp*, **51785**, 10.3791/51785 (2014). 10.3791/51785 PMC4672952 25286313

[52] Seibenhener, M. L. & Wooten, M. C. Use of the Open Field Maze to measure locomotor and anxiety-like behavior in mice. *J Vis Exp*, e52434, 10.3791/52434 (2015). 10.3791/52434 PMC4354627 25742564

[53] Coronas-Samano G, Baker KL, Tan WJ, Ivanova AV, Verhagen JV (2016). Fus1 KO mouse as a model of oxidative stress-mediated sporadic Alzheimer’s disease: circadian disruption and long-term spatial and olfactory memory impairments. Front Aging Neurosci.

