# Supplementary material for: Decreased dopamine in striatum and difficult locomotor recovery from MPTP insult after exposure to radiofrequency electromagnetic fields
Source: Sci Rep. 2019 Feb 4;9:1201. doi: 10.1038/s41598-018-37874-z (PMC6362053; doi:10.1038/s41598-018-37874-z)

**Decreased dopamine in striatum and difficult locomotor recovery from MPTP insult after exposure to radiofrequency electromagnetic fields**

Ju Hwan Kim^1^, Choong-Hyun Lee^2^, Hyung-Gun Kim^1^, and Hak Rim Kim^1*^

^1^Department of Pharmacology, College of Medicine,

^2^Department of Pharmacy, College of Pharmacy, Dankook University, Cheonan, Chungnam, Republic of Korea

Running title: Alteration of synaptic vesicles in the striatum by RF-EMF

*Correspondence: Hak Rim Kim, Ph.D.

Department of Pharmacology

College of Medicine, Dankook University

119 Dandaero, Cheonan,

Chungnam, 31116 ROK

Tel: +82-41-550-3935

Email: [hrkim@dankook.ac.kr](mailto:hrkim@dankook.ac.kr)

**Supplementary information**

**Microarray analysis**

Total RNA was extracted from the striatum of sham-treated and RF-EMF-treated mice. Microarray was performed twice with different batches of RF-EMF exposure experiments at different times (n = 5). In addition, synthesized cDNA in each batch was pooled together from the mice of each condition. cDNA was then analyzed by whole genome microarray using Nexbio (Deajeon, South Korea) to investigate the differentially expressed genes (DEGs). RNA quality was assessed using Bioanalyzer 2100 (Agilent Technologies, Santa Clara, CA). RNA QC Pass standard (acceptable RNA quality) was the rRNA ratio (28S/18S) > 1.8, % of total area (18S + 28S) > 50%, and the RNA integrity number (RIN) > 7. Cyanine 3-labeled complementary DNA (cDNA) was generated using a low RNA input linear amplification kit with 0.5 μg of total RNA. An 8 x 60 K SurePrint G3 mouse gene expression hybridization kit (Agilent Technologies) was used. Finally, DEGs were analyzed using a GeneSpring GX 12 apparatus (Agilent Technologies). A significant number of genes were differentially affected. Transcriptional profile by microarray analysis indicated that expression level of synapsin I/II/III genes was significantly decreased in the striatum after RF-EMF exposure (Table S1). Importantly, the results revealed that the basal level of synapsin III genes was more than 100 times lower compared to the synapsin I/II levels (Table S1).

**Table S1. Microarray analysis of expression synapsin in the striatum of mice after exposure to radiofrequency (RF)-electromagnetic field for 12 weeks**

| ***NCBI accession no.*** | ***Gene Symbol*** | ***Gene Name*** | ***Basal level of gene*** | | ***Fold change*** | ***p-value*** |
| --- | --- | --- | --- | --- | --- | --- |
|  |  |  | ***Sham*** | ***RF*** |  |  |
| NM_013680 | Syn1 | synapsin I | 32980.4 | 26723.1 | -0.38 | 0.003 |
| NM_001111015 | Syn2 | synapsin IIa | 1126.4 | 904.1 | -0.40 | 0.010 |
| NM_013681 | Syn2 | synapsin IIb | 31342.1 | 28117.7 | -0.23 | 0.010 |
| NM_013722 | Syn3 | synapsin IIIa | 321.8 | 224.0 | -0.60 | 0.001 |
| XM_006513703 | Syn3 | synapsin IIIf | 31.9 | 30.2 | ㅡ | ㅡ |
| NM_001164495 | Syn3 | synapsin IIIb | 2.7 | 2.8 | ㅡ | ㅡ |

**MPTP injections rapidly decrease expression level of tyrosine hydroxylase (TH) in the mouse striatum**

To explore the expression levels of TH following MPTP administration, we immunoblotted and quantified the total expression levels of TH in the striatum in both sham-exposed and RF-EMF exposed mice after MPTP injection (20 mg/kg, s.c.) (Fig. S1). The expression levels of TH in the striatum were significantly decreased up to around 80% by the systemic administration of MPTP (20 mg/kg, s.c.) in both conditions, compared to the RF-EMF-exposed mice striatum before MPTP treatment. However, the level of TH in the striatum following MPTP treatment was further decreased more than 20% in the RF-EMF exposed mice (0.2021 ± 0.02765) compared to the sham-exposed mice (0.2421 ± 0.02070).





**Fig S1. A western blotting analysis of TH expression in the striatum of sham or radiofrequency-electromagnetic field (RF-EMF) exposed mice after MPTP administration.** The TH expression were measured in the striatum before/after MPTP treatment. The band densities of TH expression were normalized to the positive control level, which used the RF-EMF exposed mouse striatum before MPTP treatment. Each bar shows mean ± SEM. Statistical significance was evaluated using two-tailed unpaired Student's *t*-test (**P* < 0.05, ***P* < 0.01, n=4).

**Fig S2. Summary of experimental design for behavioral tests.** Mice were exposed to sham or RF-EMF for 12 weeks and treated with 20 mg/kg MPTP for 3 days every 14:00 o’clock followed by one MPTP-free day and examined the locomotor activity and basic motor activity for 5 days. At the end of behavioral test of day 4, animals were euthanized for brain sample collection (n=8).


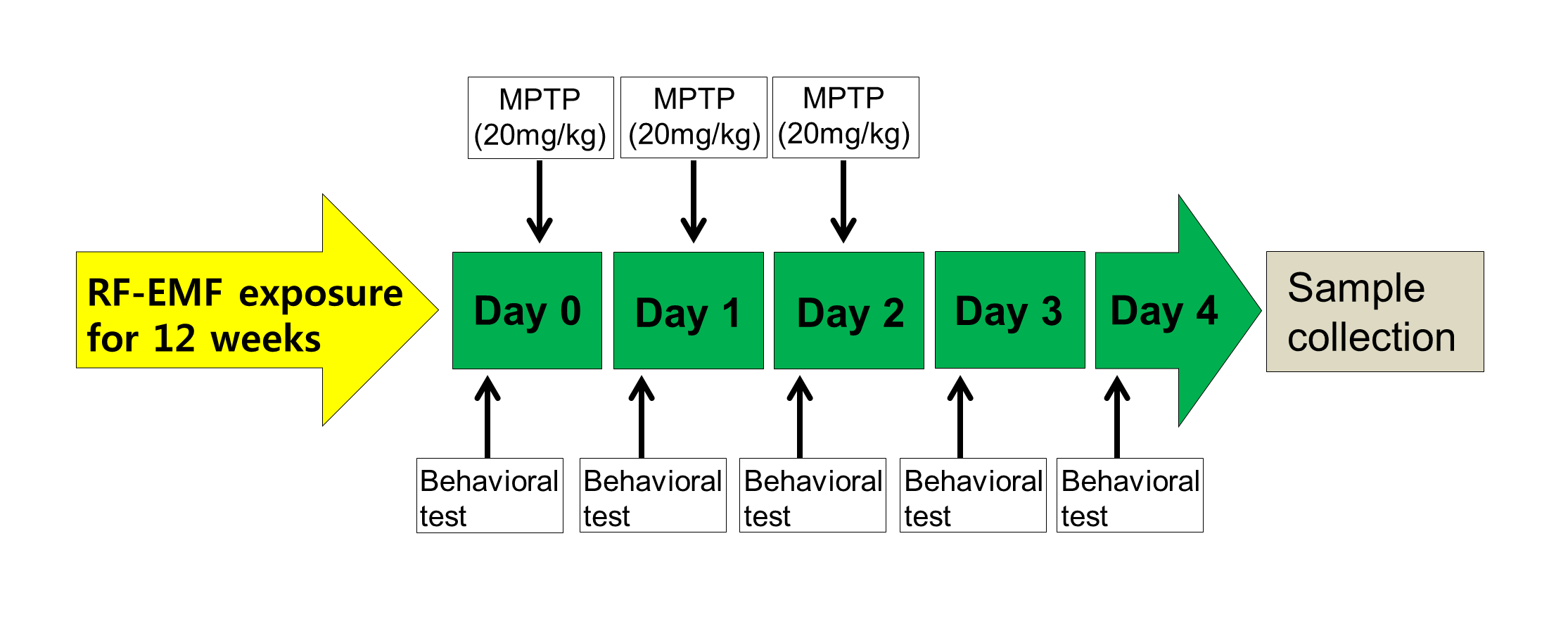

Supplement: Supplementary file 1 — Decreased dopamine in striatum and difficult locomotor recovery from MPTP insult after exposure to radiofrequency electromagnetic fields [file 41598_2018_37874_MOESM1_ESM.docx]
